# A multivariate analysis of genomic polymorphisms: prediction of clinical outcome to 5-FU/oxaliplatin combination chemotherapy in refractory colorectal cancer

**DOI:** 10.1038/sj.bjc.6601975

**Published:** 2004-06-22

**Authors:** J Stoehlmacher, D J Park, W Zhang, D Yang, S Groshen, S Zahedy, H-J Lenz

**Affiliations:** 1 Department of Hematology and Oncology, University of Hamburg, University Hospital, Hamburg 20247, Germany; 2 Department of Medical Oncology, University of Southern California/Norris Comprehensive Cancer Center, Keck School of Medicine, 1441 Eastlake Avenue, Suite 3456, Los Angeles, CA 90033, USA; 3 Department of Preventive Medicine, University of Southern California/Norris Comprehensive Cancer Center, Keck School of Medicine, Los Angeles, CA 90033, USA

**Keywords:** polymorphism, pharmacogenetics, platinum therapy, colorectal cancer

## Abstract

In this marker evaluation study, we tested whether distinct patterns of functional genomic polymorphisms in genes involved in drug metabolic pathways and DNA repair that predict clinical outcome to 5-fluorouracil (5-FU)/oxaliplatin chemotherapy in patients with advanced colorectal cancer could be identified. Functional polymorphisms in DNA-repair genes XPD, ERCC1, XRCC1, XPA, and metabolising genes glutathione *S*-transferase GSTP1, GSTT1, GSTM1, and thymidylate synthase (TS) were assessed retrospectively in 106 patients with refractory stage IV disease who received 5-FU/oxaliplatin combination chemotherapy, using a polymerase chain reaction-based RFLP technique. Favourable genotypes from polymorphisms in XPD-751, ERCC1-118, GSTP1-105, and TS-3′-untranslated region (3′UTR) that are associated with overall survival were identified. After adjustment for performance status, the relative risks of dying for patients who possessed the unfavourable genotype were: 3.33 for XPD-751 (*P*=0.037), 3.25 for GSTP1-105 (*P*=0.072), 2.05 for ERCC1-118 (*P*=0.037), and 1.65 for TS-3′UTR (*P*=0.091) when compared to their respective beneficial genomic variants. Combination analysis with all four polymorphisms revealed that patients possessing ⩾2 favourable genotypes survived a median of 17.4 months (95% confidence interval (CI): 9.4, 26.5) compared to 5.4 months (95% CI: 4.3, 6.0) in patients with no favourable genotype. Patients who carried one favourable genotype demonstrated intermediate survival of 10.2 months (95% CI: 6.8, 15.3; *P*<0.001). Polymorphisms in the TS-3′UTR and GSTP1-105 gene were also associated with time to progression. After adjustment for performance status, patients with an unfavourable TS-3′UTR genotype had a relative risk of disease progression of 1.76 (*P*=0.020) and those with the unfavourable GSTP1-105 genotype showed a relative risk of progression of 2.00 (*P*=0.018). The genomic polymorphisms XPD-751, ERCC1-118, GSTP1-105, and TS-3′UTR may be useful in predicting overall survival and time to progression of colorectal cancer in patients who receive 5-FU/oxaliplatin chemotherapy. These findings require independent prospective confirmation.

Colorectal cancer is the third most common cause of death from cancer in the United States (ACS, 2004). Despite improved screening methods, a significant proportion of cases are diagnosed in the advanced stages where chemotherapy either in the adjuvant or palliative setting is necessary. While chemotherapy has shown clearly to improve survival, the objective responses of the various drugs range between 10 and 50%, either as single agents or in combination. Furthermore, the choice of optimal chemotherapy is complicated by a wide interpatient variability of drug response and host toxicity.

Differences in drug transport, metabolism, signalling, and cellular response pathways, all contribute to the observed diversity of patients' reactions (Evans and Relling, 1999). A growing body of evidence suggests that functional genomic polymorphisms in drug target genes (Gebhardt *et al*, 1999; Lima *et al*, 1999), metabolising enzymes (Ando *et al*, 2000), and DNA-repair enzymes (Park *et al*, 2001) may have important implications for drug efficacy. One of the remaining challenges is to evaluate whether these pharmacogenetic variations are useful in predicting drug response, toxicity, and survival to specific chemotherapy regimens. Therefore, determination of significant associations between genomic polymorphisms and defined clinical end points (eg survival, response, toxicity) may improve the prediction of treatment success and thereby the tailoring of chemotherapy.

Fluoropyrimidines remain widely used in the treatment of colorectal cancer. High levels of their target enzyme thymidylate synthase (TS) have been repeatedly correlated with resistance to the agent and poorer clinical outcome (Johnston *et al*, 1995; Leichman *et al*, 1995; Aschele *et al*, 2002). A polymorphic 28-bp tandem repeat polymorphism in the regulatory region of the TS gene is associated with TS expression (Horie *et al*, 1995; Kawakami *et al*, 1999; Kawakami *et al*, 2001; Pullarkat *et al*, 2001) and toxicity to 5-fluorouracil (5-FU) chemotherapy (Pullarkat *et al*, 2001). Several groups have demonstrated that this polymorphism may also have the potential to segregate responders from nonresponders to 5-FU in both metastatic and adjuvant first-line chemotherapies (Etienne *et al*, 2002; Iacopetta *et al*, 2001; Marsh *et al*, 2001; Pullarkat *et al*, 2001; Villafranca *et al*, 2001). Its predictive value in combination and second-line chemotherapy is unknown. Moreover, the impact of a recently identified polymorphic site in the 3′-untranslated region (3′UTR) of the TS gene that may alter gene transcription (Ulrich *et al*, 2000) on clinical outcome in 5-FU-treated patients is unclear at this point.

The introduction of the platinum compound oxaliplatin in the treatment of colorectal cancer has increased response rates up to 25% even in heavily pretreated relapsing patients when administered in combination with 5-FU (Bleiberg, 1996). A randomised phase III study showed superiority of oxaliplatin and 5-FU combination therapy in refractory patients compared to either treatment alone, in terms of response rate, time to progression, and tumour-related symptoms (Rothenberg *et al*, 2003). Most recently, oxaliplatin has been approved in the US as first- and second-line therapy in combination with 5-FU for the treatment of metastatic colorectal cancer. Predictive markers for clinical outcome to this platinum agent may help to identify prospectively those patients who are more likely to benefit from the treatment.

Resistance to platinum agents has been attributed to enhanced tolerance to platinum DNA adducts, decreased drug accumulation, or enhanced DNA repair (Raymond *et al*, 1998). Proteins of the nucleotide excision repair (NER) pathway, in particular, are thought to play a key role in the repair of DNA damage caused by platinum compounds. For example, low gene expression levels of the NER protein ERCC1 were associated with superior response to 5-FU/cisplatin (Metzger *et al*, 1998) in gastric cancer and 5-FU/oxaliplatin in colorectal cancer (Shirota *et al*, 2001). Additional important players of the NER pathway include the Xeroderma pigmentosum group A and D (XPA and XPD) genes. Relevant genomic polymorphisms have been described in all genes of these NER pathway participants and preliminary data support the hypothesis that a prediction of drug response to platinum may be possible based on the analysis of their genotypes (Yu *et al*, 1997; Dybdahl *et al*, 1999; Butkiewicz *et al*, 2000; Lunn *et al*, 2000; Park *et al*, 2001; Spitz *et al*, 2001).

Glutathione *S*-transferase P1 (GSTP1) is a member of a superfamily of dimeric phase II metabolic enzymes that play an important role in the cell defense system. It is involved in platinum detoxification and is highly overexpressed in human colorectal tumours (Moscow *et al*, 1989). An activity altering polymorphism in GSTP1 has already been identified as a prognostic marker in oxaliplatin treatment (Stoehlmacher *et al*, 2002). However, its predictive value for drug response and time to progression has not yet been demonstrated. Polymorphisms in GSTT1 and M1 genes that affect GST activity have also been described (London *et al*, 2000).

In the current study, we examine a panel of 10 genetic polymorphisms within eight genes (TS, XPD, XPA, ERCC1, XRCC1, GSTP1, GSTM1, GSTT1) involved in the metabolism and detoxification of 5-FU and oxaliplatin as well as genes of the DNA-repair complex. We tested the hypothesis whether these polymorphisms, alone or in combination, may have the potential to predict clinical response, time to progression, and overall survival in advanced refractory colorectal patients treated with combination 5-FU/oxaliplatin chemotherapy.

## PATIENTS AND METHODS

### Subjects

Subjects included in this analysis were selected from a cohort of patients with metastatic colorectal cancer who were enrolled and treated on a compassionate use protocol of oxaliplatin plus 5-FU (protocol number 3C-98-3) for patients who had previously received chemotherapy for the treatment of the metastatic disease. Patients' performance status was classified according to Eastern Cooperative Oncology Group (ECOG) Criteria (Oken *et al*, 1982). Patients with an ECOG status >2 were not eligible for this study. All patients signed an informed consent prior to entering the study. This study was conducted at the University of Southern California/Norris Comprehensive Cancer Center and was approved by the Institutional Review Board of the University of Southern California for Medical Sciences. This protocol was opened for accrual in November 1998 and was closed in May 2001. A total of 263 patients were registered on the protocol. A blood and tissue collection protocol to establish the clinical significance of genetic polymorphisms in gastrointestinal cancer (OS-99-10) was opened almost 1 year after (September 1999) the opening of the 3C-98-3 protocol. Specimens from 123 patients were available for genotypic analysis.

Prior to the study described in this manuscript, we had studied the XRCC1 polymorphisms in 61 patients (Stoehlmacher *et al*, 2001), XPD polymorphisms in 73 patients (Park *et al*, 2001), and GST polymorphisms in 107 patients (Stoehlmacher *et al*, 2002). For the purpose of the current study, only patients with genotypic information in at least seven out of the 10 polymorphisms were included. When comparing these 106 patients to the 157 patients who were treated on the protocol, but not included in the current report, there were no statistically significant differences in age (*P*-value for *χ*^2^ test=0.46), race (*P*-value for *χ*^2^ test=0.55), or overall survival (*P*-value for the log-rank test=0.45). A higher percentage (75%) of patients in the current report were male compared to those (61%) who were not included (*P*-value for *χ*^2^ test=0.018).

### Clinical evaluation and response criteria

All patients enrolled in protocol 3C-98-3 had previously treated advanced colorectal tumours, which had progressed following treatment with a fluoropyrimidine-based regimen. In addition, 79% (84 of 106) of patients had also received an additional second-line treatment with irinotecan (CPT-11). Once enrolled on study, the majority (93%) of participants received the following combination therapy regimen: 130 mg m^−2^ oxaliplatin every 3 weeks and weekly continuous infusion 5-FU (200 mg m^−2^ day^−1^); one patient received 130 mg m^−2^ oxaliplatin every 3 weeks with daily bolus 5-FU; and six patients received 85 mg m^−2^ oxaliplatin every 3 weeks with weekly 5-FU.

For patients with measurable disease, responders to therapy were classified as those patients whose tumour burden (the sum, over all measurable lesions, of the products of the largest diameter and its perpendicular diameter) decreased by 50% or more for at least 6 weeks. CT imaging for response was performed every 6 weeks. For patients with evaluable but nonmeasurable disease, responders were classified as those whose tumour and all evidence of disease disappeared (a complete response, CR). Progressive disease was defined as 25% or more increase in tumour burden (compared to the smallest measurement) or the appearance of new lesions. Patients, who did not experience a response and did not progress within the first 12 weeks following start of 5-FU/oxaliplatin, were classified as having stable disease.

### Genotyping

Genomic DNA was extracted from 200 *μ*l whole blood using the QiaAmp kit (Qiagen, Valencia, CA, USA). All samples were performed using a polymerase chain reaction (PCR)–RFLP technique. After restriction enzyme analysis PCR fragments were visualised in a 2.5–3% agarose gel.

The assays for the polymorphisms in TS-5′UTR, TS-3′UTR, GSTP1-105, GSTM1, GSTT1, XRCC1-399, XPD-751, XPD-156, and XPA-5′ were performed as described previously (Horie *et al*, 1995; Arand *et al*, 1996; Harries *et al*, 1997; Yu *et al*, 1997; Dybdahl *et al*, 1999; Butkiewicz *et al*, 2000; Lunn *et al*, 2000; Ulrich *et al*, 2000). Primer sequences, restriction enzymes, and references for the analyses are given in Table 1

**Table 1:** Primer sequences and restriction enzymes

**Gene**	**Primers**	**Restriction enzyme**	**Polymorphism (localisation)**	**Significance**	**Reference**
*GSTP1-105*					
[F]	5′-ACCCCAGGGCTCTATGGGAA-3′		Ile → Val	Activity ↓	Harries *et al*
[R]	5′-TGAGGGCACAAGAAGCCCCT-3′	*Bsm*AI	(Exon 5)		
*GSTM1*					
[F]	5′-GAACTCCCTGAAAAGCTAAAGC-3′		Deletion	Activity abolished	Arand *et al*
[R]	5′-GTTGGGCTCAAATATACGGTGG-3′	—			
*GSTT1*					
[F]	5′-TTCCTTACTGGTCCTCACATCTC-3′	—	Deletion	Activity abolished	Arand *et al*
[R]	5′-TCACCGGATCATGGCCAGCA-3′				
*XRCC1-399*					
[F]	5′-TTGTGCTTTCTCTGTGTCCA-3′		Arg → Gln	DNA-repair	Lunn *et al*
[R]	5′-TCCTCCAGCCTTTTCTGATA-3′	*Msp*I	(Exon 10)	activity ↓	
*TS-5′*					
[F]	5′-GTGGCTCCTGCGTTTCCCCC-3′		28 bp repeat	Change of expression	Horie *et al*
[R]	5′-GCTCCGAGCCGGCCACAG-GCATGGCGCGG-3′		(5′-region)		
		—			
*TS-3′UTR*					
[F]	5′-CAAATCTGAGGGAGCTGAGT-3′		6 bp deletion	Unknown	Ulrich *et al*
[R]	5′-CAGATAAGTGGCAGTACAGA-3′	*Dra*I	(3-UTR)		
*XPD-751*					
[F]	5′-CCTCTCCCTTTCCTCTGTTC-3′		Lys → Gln	DNA repair	Lunn *et al*
[R]	5′-CAGGTGAGGGGGACATCT-3′	*Mbo*II	(Exon 23)	Cancer risk	
*XPD-156*					
[F]	5′-TGGAGTGCTATGGCACGATCTCT-3′		C → A	Association with	Dybdahl *et al*
[R]	5′-CCATGGGCATCAAATTCCTGGGA-3′	*Tfi*I	(Exon 6)	cancer risk	
*XPA-5′*					
[F]	5′-TCAGAAAGGCCGCTGGGT-3′		A → G	Unknown	Butkiewicz *et al*
[R]	5′-CATATCACCCCATTGTGAATG-3′	*Msp*I	(5′-region)		
*ERCC1-118*					
[F]	5′-GCAGAGCTCACCTGAGGAAC-3′		C → T	?Change of	Yu *et al*
[R]	5′-GAGGTGCAAGAAGAGGTGGA-3′	*BsrD*I	(Exon 118)	expression	

TS=thymidylate synthase; UTR=untranslated region; GSTP=glutathione *S*-transferase.

. The PCR reaction volume was 50 *μ*l. The protocol for simultaneous analysis of GSTT1 and GSTM1 was modified as follows: the primers (GSTM1, GSTT1, and albumin) were at the final concentration of 50 pmol per 50 *μ*l PCR reaction, the polymerase used was *rTaq* (Pharmacia, Piscataway, NJ, USA).

The PCR conditions for the ERCC1-118 assay were as follows: 95°C for 5 min; 40 cycles of 95°C for 1 min, annealing at 65°C for 1 min, 72°C for 1 min; and then 72°C for 7 min. The RFLP analysis of the resultant 208-bp fragment led to C/C (208 bp), C/T (208, 128, 80 bp), and T/T (128, 80 bp) genotypes.

### Statistical analysis

Initially, objective tumour response, time to progression, and overall survival were the end points considered in this analysis. Reliable toxicity data were not available in this retrospective study. Owing to the fact that in this series only nine of 101 patients who were evaluable for tumour response experienced an objective tumour response to chemotherapy, no further analysis of tumour response and its association with genetic polymorphisms was undertaken. Survival was calculated from the time that a patient started treatment until the last follow-up or death from any cause; patients who were alive at the last follow-up were censored at that time. Time to progression was calculated from the time that a patient started treatment until taken off study due to disease progression. Patients who were taken off study or who died prior to progression were censored at the time that they were taken off study. The log-rank test (Miller, 1981) and Kaplan–Meier plots (Kaplan and Meier, 1958) were used to evaluate the association of overall survival and time to progression with each of the following baseline prognostic factors: histology (well or moderate *vs* poor differentiation), side of tumour (right *vs* left), number of metastatic sites (1, 2, 3, or more), performance status (ECOG 0 or 1 *vs* 2), ethnicity (Caucasian, Hispanic, Black, and Asian), age at enrollment of study (⩽50, 51–60, and >60 years), and gender.

The association of each polymorphism with survival and time to progression was analysed singly using Kaplan–Meier plots, the log-rank test, the relative risk ratio, and its associated 95% confidence interval (CI) (Pike, 1972; Bernstein *et al*, 1981)were calculated. Contingency tables and Fisher's exact test (Metha and Patel, 1983) were used for the categorical variables to evaluate the association of the polymorphism and baseline data and the response to chemotherapy. Statistical Analysis Systems (SAS), version 8.2 (SAS Institute, Cary, NC, USA), Epilog (Epicenter, Pasadena, CA, USA) software were used for the analyses.

In the initial univariate survival analyses, ECOG performance status was the only clinical variable significantly associated (at the 0.05 level) with clinical outcome (survival and time to progression). Therefore, for the multivariate analyses, ECOG performance status was included as a stratification variable.

An internal validation analysis using bootstrapping was performed to evaluate the choice of variables selected in the final multivariate model. First, 500 bootstrap samples were generated from the original sample. Each bootstrap sample consisted of 106 observations drawn from the original data set using simple random sampling with replacement (Altman and Andersen, 1989). Second, univariate survival analysis (the log-rank test) was conducted for each of seven baseline prognostic factors and each of 10 genomic polymorphisms in each of the 500 bootstrap samples. The percentage of selection of variables for multivariable Cox's models based on the log-rank test (*P*<0.05) was calculated. The variables that were selected by more than half of the analyses of the bootstrap samples were entered in the final model (Chen and George, 1985; Altman and Andersen, 1989).

It should be noted that in this evaluation study consisting of 106 patients and 66 events, the possibility also exists that moderate patterns and associations (e.g. relative risks of 1.5–2.0) may not result in ‘statistically significant’ trends. For that reason, Table 4 has been presented in its entirety, with results for all 10 polymorphisms studied.

## RESULTS

### Patients

A total of 106 patients, consisting of 79 men (75%) and 27 women (25%) with a median age of 60 years (range: 24–84 years), were evaluated in this study. There were 75 (71%) Caucasians, 14 (13%) Hispanics, 11 (10%) Asians, and six (6%) African-American study participants. The genotype assays XPD-751, ERCC1-118, and GSTP1-105 were successful for 106 patients. Results from the remaining assays could be obtained as follows: XRCC1-399 and TS-5′UTR: 105 patients; XPD-156: 103 patients; TS-3′UTR, GSTT1, and GSTM1: 102 patients; and XPA-5′ 93 patients.

Of particular interest was the distribution of the polymorphisms within the four racial/ethnic subgroups of patients included in this series. Of the 10 sets of polymorphism studies, three (XPD-751, ERCC1-118, and TS-3′UTR) had distributions that varied across the four racial/ethnic subgroups (Table 2

**Table 2:** Association of genomic polymorphisms with ethnicity

		**Ethnicity**								
		**Caucasian**		**Hispanic**		**Black**		**Asian**		
**Factors**	**No. of patients**	***N***	**%**	***N***	**%**	***N***	**%**	***N***	**%**	P-value*
*XPD-751*										<0.001
Lys/Lys	40	19	25	8	57	4	67%	10	91	
Lys/Gln	53	46	61	4	29	2	33%	1	9	
Gln/Gln	13	11	14	2	14	0	0%	0	0	
*XPD-156*										0.14
A/A	14	12	16	1	8	0	0%	1	9	
C/A	59	43	58	7	54	1	17%	8	73	
C/C	30	19	26	5	38	5	83%	2	18	
Unknown	3	2		1						
*XPA-5′*										0.14
G/G	24	18	27	4	33	3	60%	0	0	
A/G	53	37	56	7	58	2	40%	7	64	
A/A	16	11	17	1	8	0	0%	4	36	
Unknown	13	10		2		1				
*ERCC1-118*										0.002
C/C	30	15	20	6	43	6	100	4	36	
C/T	45	34	45	5	36	0	0	6	55	
T/T	31	27	36	3	21	0	0	1	9	
*XRCC1-399*										0.63
Arg/Arg	44	29	39	5	36	3	50	7	64	
Gln/Arg	51	39	52	8	57	2	33	3	27	
Gln/Gln	10	7	9	1	7	1	20	1	9	
Unknown	1	1								
*TS-3′UTR*										0.030
+6BP/+6BP	37	31	42	4	31	1	17	1	9	
+6BP/−6BP	52	35	48	9	69	3	50	6	55	
-6BP/−6BP	13	7	10	0	0	2	33	4	36	
Unknown	4	3		1						
*TS-5′UTR*										0.25
3R/3R	43	30	39	4	31	1	17	8	73	
2R/3R	46	34	45	7	54	3	50	3	27	
2R/2R	16	12	16	2	15	2	33	0	0	
2/4	1			1						
*GSTP1-105*										0.61
Val/Val	10	7	9	1	7	1	17	1	9	
Ile/Val	47	35	46	6	43	4	67	3	27	
Ile/Ile	49	34	45	7	50	1	17	7	64	
*GSTT1*										1.00
Positive	75	53	72	10	77	4	80	8	73	
Negative	27	21	28	3	23	1	20	3	27	
Unknown	4	2		1		1				
*GSTM1*										0.14
Positive	54	39	53	9	69	4	80	3	27	
Negative	48	35	47	4	31	1	20	8	73	
Unknown	4	2		1		1				

GSTP=glutathione *S*-transferase; TS=thymidylate synthase; UTR=untranslated region.

* *P*-value is based on Fisher's exact test.

). No clear patterns for significant associations between any of these polymorphisms and any of the other demographic (gender), clinical (performance status, localisation of the tumour, and number of metastases), or pathological characteristics (tumour differentiation) were observed. Of these 50 comparisons, the following four associations generated *P*-values less than 0.05 (based on Fisher's exact test): gender with TS-5′UTR where more females had the 3R/3R genotype (*P*=0.031); number of metastatic sites with XPA-5′ (*P*=0.015) and TS-3′UTR (*P*=0.046); and ECOG performance status and XRCC1-399 (*P*=0.009).

### Demographic, clinical, and pathological characteristics and clinical outcome

With a median follow-up of 11.4 months (range: 3.3–30.9 months) for the 106 patients of the study, the median survival time was 10.2 months (95% CI: 7.0, 13.2 months). The median time to progression was 4.7 months (95% CI: 4.2, 5.9 months).

Age, ethnicity, and sex were not associated with patient survival. Performance status was significantly associated with survival, with patients with a performance status ECOG=2 showing an increased risk of dying of 3.46 (95% CI: 1.99, 6.01; *P*<0.001) when compared to those with (ECOG 0-1) upon protocol entry. Patients with a right-sided tumour tended to have an increased risk of dying sooner (RR=1.55, *P*=0.077, Table 3

**Table 3:** Association between clinical and pathological characteristics of study participants and survival and progression after 5-FU/oxaliplatin chemotherapy

		**Survival**		**Progression^a^**	
**Factors**	**No. of patients**	**Relative risk of dying^b^ and 95% CI**	***P*-value^c^**	**Relative risk of progression^a^ and 95% CI**	***P*-value^c^**
*Age (years)*			0.70		0.54
>60	50	1		1	
51–60	26	1.27 (0.69, 2.35)		1.03 (0.60, 1.77)	
⩽50	30	1.16 (0.64, 2.08)		1.30 (0.79, 2.13)	
					
*Gender*			0.72		0.50
Male	79	1		1	
Female	27	1.10 (0.65, 1.87)		0.84 (0.51, 1.40)	
					
*Ethnicity*			0.26		0.56
Caucasian	75	1		1	
Hispanic	14	0.65 (0.30, 1.38)		1.00 (0.51, 1.97)	
Black	6	0.46 (0.14, 1.47)		0.70 (0.25, 1.96)	
Asian	11	0.55 (0.20, 1.52)		1.47 (0.75, 2.90)	
					
*Histology*			0.058		0.69
Moderate/well	77	1		1	
Poor	16	1.85 (0.93, 3.66)		1.13 (0.61, 2.11)	
Unknown	13				
					
*Side of tumour*			0.077		0.51
Left	71	1		1	
Right	34	1.55 (0.93, 2.60)		1.16 (0.74, 1.82)	
Unknown	1				
					
*Number of metastatic sites*			0.95		0.60
1	46	1		1	
2	41	0.94 (0.57, 1.63)		0.81 (0.51, 1.30)	
3+	19	0.91 (0.46, 1.78)		0.79 (0.43, 1.47)	
					
*Performance status*			<0.001		0.001
0–1	78	1		1	
2	28	3.46 (1.99, 6.01)		2.15 (1.30, 3.57)	

5-FU=5-fluorouracil; CI=confidence interval.

a Time to progression was calculated from the time that patient started treatment until the patient was taken off study. If the relative risk is greater than 1, the relative risk can be thought as the average increased risk of progression at any point in time compared to the reference group. The group with the ratio equal to 1 is the reference group.

b If the relative risk is greater than 1, the relative risk can be thought as the average increased risk of dying at any point in time compared to the reference group. The group with the ratio equal to 1 is the reference group.

c *P*-value based on log-rank test.

) if compared to left-sided tumours, while the 16 patients with a poorly differentiated tumour also tended to have a shorter survival (*P*=0.058). Bootstrap analysis confirmed that ECOG status was selected by over 99% of the bootstrap samples as the only baseline prognostic variable significantly associated with survival.

In addition, patients with a performance status ECOG=2 showed an increased risk of progressing, of 2.15 (95% CI: 1.30, 3.57; *P*=0.001) when compared with patients who showed a superior performance status (ECOG 0–1) upon protocol entry. Age, ethnicity, sex, tumour histologic grade, and side of tumour were not associated with time to progression in this series of patients.

### Association between the genetic polymorphisms and survival

By univariate analysis, the XPD-751, ERCC1-118, and GSTP1-105 polymorphisms were significantly associated with survival among study participants (Table 4

**Table 4:** Association between polymorphisms in the genes of TS, ERCC1, XPD, XPA, XRCC1, GSTP1, GSTT1, and GSTM1 and survival and progression after 5-FU/oxaliplatin chemotherapy

		**Survival**		**Progression^a^**	
**Factors**	**No. of patients**	**Relative risk of dying^b^ and 95% CI**	***P-*value^c^**	**Relative risk of progression^a^ and 95% *CI***	***P-*value^c^**
*XPD-751*			0.049		0.76
Lys/Lys	40	1		1	
Lys/Gln	53	1.87 (1.06, 3.31)		1.13 (0.72, 1.78)	
Gln/Gln	13	2.44 (1.09, 5.44)		1.25 (0.59, 2.67)	
*XPD-156*			0.88		0.64
A/A	14	1		1	
C/A	59	1.22 (0.55, 2.75)		0.81 (0.44, 1.49)	
C/C	30	1.18 (0.49, 2.82)		0.73 (0.36, 1.48)	
Unknown	3				
*XPA-5′*			0.76		0.61
G/G	24	1		1	
A/G	53	0.88 (0.47, 1.62)		1.24 (0.70, 2.18)	
A/A	16	1.13 (0.49, 2.45)		1.37 (0.66, 2.84)	
Unknown	13				
*ERCC1-118*			0.021		0.51
C/C	30	1		1	
C/T	45	2.29 (1.19, 4.41)		1.24 (0.73, 2.11)	
T/T	31	1.86 (0.91, 3.83)		1.36 (0.76, 2.41)	
*XRCC1-399*			0.50		0.97
Arg/Arg	44	1		1	
Gln/Arg	51	1.07 (0.63, 1.80)		0.95 (0.60, 1.51)	
Gln/Gln	10	1.58 (0.71, 3.55)		0.99 (0.47, 2.09)	
Unknown	1				
*TS-3′UTR*			0.72		0.22
+6BP/+6BP	37	1		1	
+6BP/−6BP	52	1.23 (0.70, 2.15)		1.49 (0.92, 2.40)	
-6BP/−6BP	13	1.18 (0.52, 2.66)		1.39 (0.70, 2.75)	
Unknown	4				
*TS-5′UTR*			0.42		0.44
3R/3R	43	1		1	
2R/3R	46	0.82 (0.49, 1.39)		0.78 (0.48, 1.26)	
2R/2R	16	1.32 (0.64, 2.75)		1.06 (0.55, 2.02)	
2/4	1				
*GSTP1-105*			0.019		0.011
Val/Val	10	1		1	
Ile/Val	47	1.82 (0.71, 4.66)		1.22 (0.56, 2.67)	
Ile/Ile	49	2.96 (1.15, 7.61)		2.13 (0.95, 4.76)	
*GSTT1*			0.29		0.93
Positive	75	1		1	
Negative	27	1.33 (0.78, 2.28)		0.98 (0.60, 1.60)	
Unknown	4				
*GSTM1*			0.59		0.58
Positive	54	1		1	
Negative	48	1.14 (0.69, 1.88)		1.13 (0.72, 1.76)	
Unknown	4				

5-FU=5-fluorouracil; CI=confidence interval; GSTP=glutathione *S*-transferase; TS=thymidylate synthase; UTR=untranslated region.

a Time to progression was calculated from the time that patient started treatment until the patient was taken off study. If the relative risk is greater than 1, the relative risk can be thought as the average increased risk of progression at any point in time compared to the reference group. The group with the ratio equal to 1 is the reference group.

b If the relative risk is greater than 1, the relative risk can be thought as the average increased risk of dying at any point in time compared to the reference group. The group with the ratio equal to 1 is the reference group.

c *P*-value based on log-rank test.

). Bootstrap analysis confirmed that ERCC1-118, GSTP1-105, and XPD-751 were selected by over half of the bootstrap samples (68–73%) as polymorphisms significantly associated with survival.

#### XPD-751

In all, 40 patients (38%) possessed the XPD-751 *Lys/Lys* genotype, 13 (12%) showed the XPD-751 *Gln/Gln* genotype, and 53 (50%) patients were heterozygous for this variant. Using the XPD-751 *Lys/Lys* group as a reference the *Gln/Gln* group showed a 2.44-fold (95% CI: 1.09, 5.44) increased risk of dying, whereas patients with the heterozygous genotype showed an intermediate relative risk of 1.87 (95% CI: 1.06, 3.31) (*P*=0.049).

#### GSTP1-105

Patients who were identified with a GSTP1-105 Ile/Ile genotype demonstrated a 2.96-fold (95% CI: 1.15, 7.61) increased risk of dying if compared with the GSTP1-105 Val/Val group. Patients with a GSTP1-105 Val/Ile genotype showed a 1.82 increase in risk of dying (95% CI: 0.71, 4.66) (*P*=0.019) compared to those with the Ile/Ile genotype. The GSTP1-105 genotype distribution was as follows: 49 (46%) GSTP1-105 Ile/Ile, 47 (44%) Val/Ile, and 10 (9%) Val/Val.

#### ERCC1-118

In terms of the ERCC1-118 polymorphism, patients with a C/C genotype showed the most favourable survival. Patients whose tumours were identified with a CT substitution for both alleles demonstrated a relative risk of dying of 1.86 (95% CI: 0.91, 3.83) compared to patients with the C/C genotype, whereas the heterozygous patients showed a 2.29-fold (95% CI: 1.19, 4.41) increase in risk (*P*=0.021). The majority of patients (45, 42%) possessed the heterozygous genotype, 30 patients (28%) showed no C → T substitution, and 31 patients (29%) demonstrated a replacement of C by T for both alleles. For the subsequent multivariable analyses, the patients with the T/T and C/T genotypes were combined and compared to the patients with the C/C genotype, for reasons of parsimony and to preserve monotonicity, since in this series there was no evidence of a difference between the T/T and C/T genotypes.

#### TS-3′UTR

The TS-3′UTR polymorphism was not significantly associated with survival in the univariate analysis. Nonetheless, because TS gene expression is known to be an important predictor of outcome for patients with colorectal cancer receiving 5-FU-based therapy, the TS polymorphisms at two sites were included in the initial multivariable model. The TS-5′UTR polymorphism displayed no association with survival or time to progression, with TS-3′UTR in the Cox model. However, when all four polymorphisms XPD-751, ERCC1-118, TS-3′UTR, and GSTP1-105 were stratified by performance status and analysed jointly in the multivariable analysis, TS-3′UTR was weakly associated with survival. A decision was made to include TS-3′UTR in the final model since it remained strongly associated with time to progression (Table 5

**Table 5:** Joint association of XPD-751, ERCC1-118, TS-3′UTR, and GSTP1-105 polymorphisms with survival and time to progression after 5-FU/oxaliplatin chemotherapy for disseminated colorectal cancer (multivariable analysis, stratified by ECOG)

		**Survival**		**Progression**	
**Factors**	**No. of patients**	**Adj. relative risk^a^**	**Adj. *P*-value^a^**	**Adj. relative risk^b^**	**Adj. *P*-value^b^**
*XPD-751*			0.037		
Lys/Lys	40	1			
Lys/Gln	53	1.50 (0.79, 2.87)			
Gln/Gln	13	3.33 (1.39, 7.99)			
					
*ERCC1-118*			0.037		
Favourable: C/C	30	1			
Unfavourable	76	2.05 (1.00, 4.20)			
					
*TS-3′UTR*			0.091		0.020
Favourable: +6BP/+6BP	37	1		1	
Unfavourable	65	1.65 (0.91, 2.99)		1.76 (1.08, 2.86)	
Unknown	4				
					
*GSTP1-105*			0.072		0.018
Val/Val	10	1		1	
Ile/Val	47	2.73 (0.88, 8.45)		0.96 (0.42, 2.21)	
Ile/Ile	49	3.25 (1.04, 10.16)		2.00 (0.85, 4.71)	

5-FU=5-fluorouracil; CI=confidence interval; GSTP=glutathione *S*-transferase; TS=thymidylate synthase; UTR=untranslated region.

a Based on Cox's proportional-hazards model, stratified by ECOG, with all four genes included for survival.

b Based on Cox's proportional-hazards model, stratified by ECOG, with TS-3′UTR and GSTP1-105 for time to progression.

) and because of its potential role in 5-FU action. As with ERCC1, the two genotypes (−6BP/−6BP and +6BP/−6BP) were grouped for reasons of parsimony and to preserve monotonicity, because of the lack of evidence of any difference.

The analyses of the polymorphisms for XPD-156, GSTT1, GSTM1, XRCC1-399, XPA-5′, and TS-5′UTR did not show significant associations between overall survival or disease progression (Table 4).

### Combined analysis XPD-751, ERCC1-118, GSTP1-105, and TS-3′UTR for survival

When XPD-751, ERCC1-118, TS-3′UTR, and GSTP1-105 were analysed jointly, stratified by ECOG performance status, XPD-751 and ERCC1-118 remained significantly associated with survival (*P*<0.05, see Table 5) and the associations of TS-3′UTR and GSTP1-105 were borderline (0.05<*P*<0.10). Bootstrap analysis confirmed that polymorphisms were selected for the final multivariable model in 46% for TS 3′ UTR, 52% for GSTP1-105, 62% for ERCC1-118, and 72% for XPD-751. Overall, the bootstrap analysis confirmed the selection of polymorphisms in the original final model.

Based on these observations, we performed a second analysis to elucidate whether a pattern of favourable genotypes could be used to determine clearcut differences of clinical outcome. For this, a favourable genotype for each polymorphism was identified and compared with the group of the remaining two unfavourable genotypes of that polymorphism. In all, 25 patients had no favourable polymorphisms, 48 patients had one favourable polymorphisms, 21 patients had exactly two, and eight patients had three favourable polymorphisms. Among the four patients with a missing TS-3′UTR assessment, one had two favourable polymorphisms and was included in the group of patients having 2+ favourable polymorphisms. In this analysis, patients who possessed two or more favourable genotypes survived a median of 17.4 months (95% CI: 9.4, 26.5) compared to patients who had no favourable genotypes and had a median survival of only 5.4 months (95% CI: 4.3, 6.0). Patients with one favourable genotype showed an intermediate median survival time of 10.2 months (95% CI: 6.8, 15.3) (*P*<0.001). Using the group of patients with two or more favourable polymorphisms as the reference group, the relative risk of dying was 2.14 (95% CI: 1.09, 4.22) and 3.75 (95% CI: 1.78, 7.89) for patients with only one or 0 favourable polymorphisms, respectively (Table 6

**Table 6:** Combined analysis of association between XPD-751, ERCC1-118, TS-3′UTR and GSTP1-105 polymorphisms and survival after 5-FU/oxaliplatin chemotherapy for disseminated colorectal cancer

**Number of favourable polymorphisms^a^**	**Patients (*N*=103)^b^**	**Relative risk (95% CI)**	**Median survival (95% CI)**	***P*-value^c^**
*Lys/Lys, C/C, Val/Val*, +*6BP*/+*6BP*				<0.001
⩾2	30	1	17.4 months (9.4, 26.5)	
1	48	2.14 (1.09, 4.22)	10.2 months (6.8, 15.3)	
0	25	3.75 (1.78, 7.89)	5.4 months (4.3, 6.0)	
Unknown	3			

5-FU=5-fluorouracil; CI=confidence interval; GSTP=glutathione *S*-transferase; TS=thymidylate synthase; UTR=untranslated region.

a Favourable genotypes of XPD-751 (*Lys/Lys*), ERCC1-118 (*C/C*), GSTP1-105 (*Val/Val*), and TS-3′UTR (+*6BP*/+*6BP*) polymorphisms.

b Combined analysis was performed for only 103 out of 106 patients. Among the four patients with a missing data of the TS-3′UTR polymorphism, one patient was included since the patient had two favourable polymorphisms (ERCC1-118 and GSTP1-105).

c Based on log-rank test.

; Figure 1

**Figure 1:**
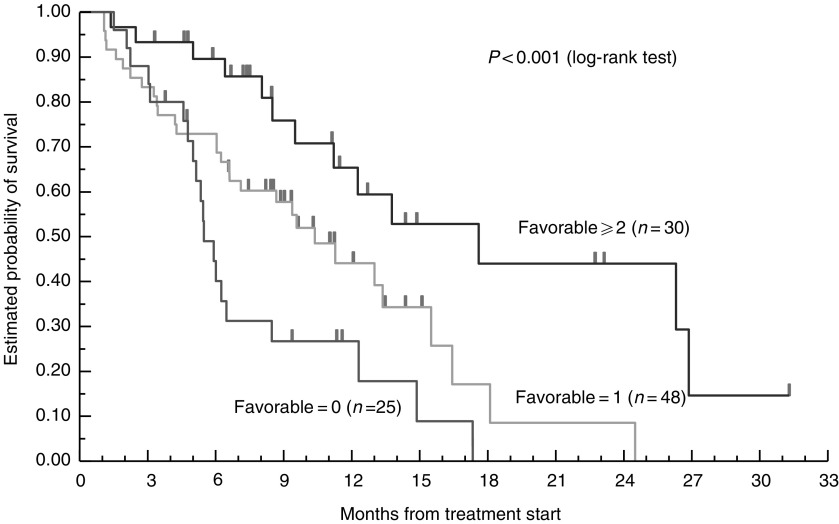
Favourable genomic polymorphism and overall survival (*n*=103).

).

### Association between time to progression and TS-3′UTR, GSTP1-105 polymorphisms

In the univariate analysis, a dose-dependent association was observed for the GSTP1-105 polymorphism, demonstrating a decreased risk of progressing for patients possessing two or one GSTP1-105 Val alleles (*P*=0.011). Using the GSTP1-105 Val/Val carriers as reference, individuals with a GSTP1-105 Ile/Ile genotype demonstrated a 2.13-fold (95% CI: 0.95, 4.76) risk for disease progression. Heterozygotes had an intermediate risk of progressing (relative risk of 1.22 (95% CI: 0.56, 2.67). Patients with one or two −6BP alleles of the TS-3′UTR genotype appeared to have a higher risk of progressing comparing to the +6BP/+6BP homozygotes, although this association did not reach statistical significance in the univariate analysis (*P*=0.22) (Table 4).

In the multivariate analysis, stratifying by performance status, the relative risk for progression was 1.76 (95% CI: 1.08, 2.86) for the TS-3′UTR +6BP/−6BP and −6BP/−6BP genotypes combined, when compared to patients with the +6BP/+6BP genotype (*P*=0.020). Using the GSTP1-105 Val/Val genotypes as reference, patients with GSTP1-105 Val/Ile genotype demonstrated a relative risk of 0.96 (95% CI: 0.42, 2.21). The risk of disease progression for the GSTP1-105 Ile/Ile genotype was 2.00 (95% CI: 0.85, 4.71) compared to the risk of the GSTP1-105 Val/Val genotype (*P*=0.018).

## DISCUSSION

In the current study, we have attempted to move beyond single gene polymorphisms to a more comprehensive pathway evaluation to identify genomic variants and patterns that may help predict clinical response and overall survival to platinum-based chemotherapy, thus ultimately leading to a more tailored approach to chemotherapy. Polymorphisms in the genes of GSTP1, ERCC1, XPD, and TS were associated with overall survival in patients with refractory advanced colorectal cancer who received 5-FU/oxaliplatin combination chemotherapy. Combination of *favourable* genotypes of these polymorphisms identified those patients who would gain the greatest survival benefit from 5-FU/oxaliplatin combination chemotherapy. In addition, the GSTP1 (univariate and multivariate analysis) and TS 3′-UTR (multivariate analysis) gene variants were associated with time to progression. Unfortunately, the low number of clinical responses in our cohort (nine out of 101 evaluable patients) limited analysis between clinical response and gene polymorphisms. However, we were able to perform time to progression analyses that may also be used as measures of chemotherapy effectiveness.

*In vitro* reports provide strong evidence that activity of members of the NER pathway, especially ERCC1, is important for the removal of DNA adducts caused by platinum compounds (Reed, 1998). ERCC1 is a highly conserved protein and its role is critical in DNA-damage recognition and DNA-strand incision. An analysis by Yu *et al* (1997) has identified a common C → T polymorphism at codon 118 of the ERCC1 gene, which results in the same amino-acid asparagine. Preliminary data by Park *et al* (2002) in colon tumours demonstrated a trend towards higher ERCC1 mRNA levels as the number of T alleles increased. Since increased gene expression of ERCC1 can lead to platinum resistance, survival benefit of C/C carriers in the current study may support these findings. However, the mechanism for this is unclear. In fact, this silent polymorphism is not part of any known regulatory binding site. Furthermore, other expression analysis in ovarian cancer cell lines do not confirm these results (Yu *et al*, 2000).

The association between the ERCC1 polymorphism and overall survival in our study was not linear. One possible explanation is that the polymorphism might be in linkage disequilibrium with another factor influencing survival to platinum-based chemotherapy within the same gene or a gene nearby, such as XPD or XRCC1. This effect might over-ride an alteration of ERCC1 gene expression by the polymorphism. A second possible explanation is that with the numbers of patients included in this series, this lack of monotonicity occurred by chance alone; analysis with an independent data set will be necessary to better characterise this association.

Another essential member of the NER pathway is the XPD gene, otherwise known as ERCC2 (Sung *et al*, 1993). Reports have shown that common polymorphisms in the XPD gene may be associated with differential DNA repair capacity (Dybdahl *et al*, 1999; Lunn *et al*, 2000; Spitz *et al*, 2001). Interestingly, studies with regard to the polymorphisms' impact on DNA repair capacity have yielded diverging results (Dybdahl *et al*, 1999; Lunn *et al*, 2000; Spitz *et al*, 2001; Chen *et al*, 2002). Recently, preliminary results from our group supported the hypothesis that the 751*Lys* allele may be associated with an impaired NER efficacy (Park *et al*, 2001). The current data indirectly confirm this hypothesis, since patients who possess one or two 751*Gln* alleles benefited less from platinum-based chemotherapy compared to 751*Lys* carriers. Although it has been posited that the proximity of the substituted polar amino acid to the poly(A) signal may be affect XPD protein function (Dybdahl *et al*, 1999), the molecular mechanism by which this polymorphism alters the efficacy of oxaliplatin in tumour cells remains unknown. A second polymorphism in ERCC2 (XPD-156) and other members of the DNA repair pathway (XPA-5 and XRCC1-399) were not found to be associated with clinical outcome.

The purpose of this study was to evaluate the impact of genomic variations on different mechanisms that alter drug efficacy. Besides changes in DNA-repair function, the rate of inactivation of the administrated drug compound may also determine its efficacy in the tumour tissue. The GST superfamily participates in the detoxification processes of platinum compounds (Ban *et al*, 1996; Goto *et al*, 1999). We have recently reported that the GSTP1 polymorphism at codon 105 may be associated with superior overall survival in colorectal cancer patients (Stoehlmacher *et al*, 2002). The current study identifies this GSTP1-105*Val* variant as a predictor for time to progression to 5-FU/oxaliplatin, as well as overall survival.

Earlier reports in human tissues demonstrated a decline in GSTP1 activity with an increasing number of GSTP1-105*Val* alleles (Watson *et al*, 1998). Our data suggest that patients with the *Val* allele might experience protracted detoxification of oxaliplatin. Thus, a prolonged exposure of the tumour to oxaliplatin may lead to an increased overall efficacy of the drug and a superior survival.

On the other hand, the deletion polymorphisms of GSTT1, and the GSTM1, which are associated with abolished enzyme activity (London *et al*, 2000), were not associated with clinical outcome. Predominant expression of the GSTP1 subclass (compared to GSTT1 and GSTM1) in colorectal epithelial and tumour tissue may explain in part this phenomenon (Moscow *et al*, 1989).

Analyses of polymorphisms in genes of DNA-repair and phase II metabolising enzymes focused on the oxaliplatin effect. Modifications of 5-FU efficacy were also evaluated by analysing genomic variants in the gene of its target molecule TS. Two common polymorphic sites within the TS gene that might alter expression levels have been described (Horie *et al*, 1995; Ulrich *et al*, 2000). Although there have been reports of four, five, and nine repeats within certain African and Asian populations (Luo *et al*, 2002; Marsh *et al*, 2000), the majority of individuals possesses either two repeats (2R) or three repeats (3R) of a 28-bp sequence.

We hypothesised that the 5′-TS polymorphism maybe predictive for clinical outcome in patients of the current study. This polymorphism was not associated with overall survival or time to progression in our cohort. One reason for this lack of prediction may be the fact that these patients received combination chemotherapy. Second, a genomic polymorphism represents a static value unable to change in response to a new situation in the tumour cell (e.g. changes in oxygen supply, chemotherapy) as it would be reflected by expression levels. Thus, a strong impact of the genomic variation on functionality is needed to keep the power of prediction, especially when other confounders (e.g. previous and combination chemotherapy) are present. Although several reports have consistently revealed an association of the 5′-TS polymorphism and clinical outcome to 5-FU, a sizeable fraction of patients with the triple repeat genotype in those studies showed low TS expression or some short-term benefit to 5-FU chemotherapy (Iacopetta *et al*, 2001; Pullarkat *et al*, 2001), thus indicating the limitations of the predictive strength of this polymorphism. Other factors (e.g. transcriptional factors, LOH in the tumour) may in addition alter the expression, and this influence cannot be accurately reflected by a genotype analysis. Most recently, our group has identified a novel single-nucleotide polymorphic change within the 5′-TS tandem repeat within the USF-1 consensus element that abolishes USF-1 binding and alters transcriptional activation (Mandola *et al*, 2003). The clinical implication of this finding is unclear at this time. We are currently engaged in a prospective confirmatory study in refractory colorectal cancer patients under platinum-based therapy where the 5′-TS tandem repeat is analysed in the context of this novel polymorphism.

At the moment, less information is available with regard to a recently identified 6-bp deletion polymorphism in the 3′UTR of the TS gene. This polymorphism might alter mRNA stability, its secondary structure, or expression as it has been demonstrated for alterations of the 3′-region in other genes (Gou *et al*, 1998; Rajagopalan and Malter, 2000). Preliminary data show that the TS-3′ UTR polymorphism may be correlated with differential TS gene expression, with 6-bp deletions leading to lower intratumoral TS mRNA (Lenz *et al*, 2002a). In our study, however, patients who possessed the deletion on one or both chromosomes were at a higher relative risk of dying and disease progression after stratification by performance status. We have no compelling mechanistic explanation for this finding at this time.

Nevertheless, these data suggest that this polymorphism maybe associated with TS functionality. Further biochemical analyses are needed to explore the significance of this polymorphism for TS function. In addition, it would be helpful to perform haplotype analyses of both TS polymorphisms in order to determine possible opposing or synergistic effects of these polymorphisms on TS expression and clinical outcome. The association between TS-5′ and TS-3′ polymorphisms, although significant (*P*=0.037, Pearson's *χ*^2^ test), did not reveal a clear pattern in our cohort.

Since the time of our analysis presented in the current report, and previously in abstract form (Lenz *et al*, 2002b), preliminary data from the first large-scale prospective pharmacogenetic study in colorectal cancer was recently presented. The aim of the study was to validate previously reported correlations between the various genomic polymorphisms (including several included in this report) and clinical response and toxicity. The pharmacogenetic element was added midway to a phase III GI intergroup trial (N9741) comparing three regimens for first-line therapy in metastatic colorectal cancer. In all, 18 genetic variants in 11 genes were assessed. The XPD*751Gln* genotype was associated with decreased clinical response to oxaliplatin-based regimen. This is consistent to our previously reported clinical correlation. Furthermore, the TS-3′UTR polymorphism was also found to be associated with clinical response and time to progression, GST polymorphisms were not associated with clinical response, and ERCC1-118 polymorphism was not evaluated. No data on overall survival were available at the time of abstract presentation (McLeod *et al*, 2003).

Significant differences in patient selection (initial metastatic *vs* refractory colorectal cancer), treatment schedules, and other factors exist between the two studies, which may or may not have contributed to the consistent or discordant results. Moreover, the N9741 pharmacogenetic data are available only in preliminary form at this time, making meaningful discussion or comparison of the data difficult. Still, these preliminary results underscore the possibility of generating a genomic profile that may help predict clinical outcome to a given chemotherapeutic regimen. This would be especially useful now that we are at a juncture where equivalent chemotherapeutic regimens with differing mechanisms of action are available for the treatment of advanced colorectal cancer (Tournigand *et al*, 2004).

To conclude, this pilot study demonstrates that a combined genotype analysis of GSTP1-105, TS-3′UTR, ERCC1-118, and XPD-751 may contribute to the selection of patients who would benefit from 5-FU/oxaliplatin chemotherapy. Our study is limited by its retrospective nature with a relatively small sample size compared to the total number of patients treated in the protocol. As stated before, the small number of responses did not allow for the identification of predictors of response, although predictors of time to progression were identified. To our knowledge, this is the first study that identifies a pharmacogenetic profile that may predict clinical outcome to platinum-based chemotherapy in advanced colorectal cancer. The resampling analysis suggests that the current study has internal validity, that is, others analysing the number of favourable polymorphisms would reach the same conclusions, and that this approach has potential and merits further investigation.
